# Re description and molecular identification of *Thalamita danae* (Stimpson 1858) (Decapoda: Brachyura: Portunidae) based on fresh material from the coastal waters, Pakistan

**DOI:** 10.1080/23802359.2018.1438855

**Published:** 2018-03-10

**Authors:** Noor Us Saher, Farah Naz, Syeda Mariam Siddiqa, Mohammad Mumtaz Khan, Mustafa Kamal

**Affiliations:** a Centre of Excellence in Marine Biology, University of Karachi, Karachi, Pakistan;; b Department of Biotechnology, University of Karachi, Karachi, Pakistan;; c Department of Microbiology, University of Haripur, Hripur, Pakistan

**Keywords:** Brachyura, systematics, molecular phylogeny, mt DNA, Pakistan coast

## Abstract

*Thalamita danae* (Stimpson1858), re-described as a new report, based on an integrative taxonomy approach combining 16S rRNA partial sequence of mitochondrial DNA and morphological analyses was used for the accurate identification of specimens. The morphological and molecular analysis provides the confirm evidence of *T. danae* in coastal waters of Pakistan. Results were confirmed by amplification of partial sequences of 16S rRNA mt-DNA gene and the sequence was searched for similarity using BLASTn (Basic Local Alignment Tool), the result showed 97% sequence similarity with partial sequences of *T. danae* Stimpson, 1858 as obtained from gene bank. The attained sequence was submitted to gene bank after confirmation of genetic and morphological similarity. High sequence similarity with accession no: FJ152165.1 indicated, that misidentification of species does not occur.

## Introduction

The family Portunidae (Rafinesque, 1815) has worldwide distribution (Hartnoll 1971; Apel and Spiridonov 1998; Ng et al. 2008) and a distinctive group of marine crabs with well representation in Southeast Asia (Wee and Ng 1995)and includes species of marked economic importance mainly genus *Scylla* and *Portunus* (Spiridonov et al. 2014).

Besides their economic and ecological significance and availability of previous taxonomic checklist (Hashmi 1963a, b; Guinot, 1969; Khan and Ahmed 1973; Tirmizi and Kazmi 1996) the molecular confirmation is still required for the Portunid fauna of Pakistani waters. Twenty species of Portunid crabs reported from coastal waters of Pakistan, pertaining to six genera (*Podophthalmus, Carcinus*, *Scylla, Portunus, Charybdis* and *Thalamita*). In previous studies, only three species of genus *Thalamita* Latreille, 1829: (*T. admete*, *T. crenata* and *T. prymna*) reported from Pakistan. Now one more species; *Thalamita danae* (Stimpson, 1858) was identified as new report based on morphological and molecular analysis.

## Materials and methods

Karachi constitutes a coastal belt of about 100 km long between the Indus delta on the southeast coast and the Hub River on the west. The examined fresh material of *Thalamita danae *originated from the rocky coast of Buleji (24°47 815°N, 67°49 280°S). After confirmation of species through sequence analysis, these specimens were again collected through handpicked method for morphological description and confirmation of species status. After taking the DNA sample and morphometric measurements: Carapace length (CL), Carapace width (CW), Cheliped length (Ch.L), to the nearest 0.1 mm, the sample preserved in ethanol 80% to be used for taxonomic identification. The species identified as *T. danae* and than verified on the basis of morphological characterstics (Yu 1979; Wee and Ng 1995; Poore and Ahyong 2004). The specimens were deposited in the Marine Reference Collection and Resource Centre (MRCC), the University of Karachi as Catalog no. BRAC 759. Images were obtained using Scanning electron microscope (model no. JOEL, JSM-6380 LV).

## Comparative material

Three species of *Thalamita* (*T. admete*, Catalog no. BRAC, 573, *T. creneta*, Catalog no. BRAC, 381 and *T. prymna* Catalog no. BRAC, 422) present in MRCC, University of Karachi.

## DNA extraction and sequence analysis

Species identification exclusively based on a partial fragment of the mt DNA 16S rRNA gene. The approximately 25 mg of tissue samples from Chela were used for the extraction of total genomic DNA by using Qiagen DNeasy Blood and Tissue Kit (Cat. no. 69504),

The polymerase chain reactions (PCR) technique was used to amplify the region of the 16S rRNA gene by using the primers 16Sar forward (5′-CGC CTG TTT ATC AAA AAC AT-3′) paired with the reverse primer 16Sbr (5′-CCG GTC TGA ACT CAG ATC ACG T-3′) (Palumbi et al. 1991; Schubart et al. 2000; Fratini et al. 2005).

The PCR amplification was performed in an Applied Biosystem 2720 thermal cycler (Applied Biosystems, Foster City, CA) and the PCR conditions applied following Lai et al. (2010).

PCR products confirmed for gene amplification through 2% agarose gels with ethidium bromide stained. Successfully amplified PCR products were purified and sequenced from the Macrogen Company (Souel, Korea) and the procured sequence was utilized for the species identification as initially searched for sequence similarity using the NCBI web link (www.ncbi.nlm.nih.gov/BLAST (Basic Local Alignment Search Tool). Sequences were aligned using the Clustal W tool, MEGA 6, MEGA Inc., Ocheyedan, IA). DNA sequence data were analyzed through applied biosystem sequence Scanner v1.0 software (SPSS, Chicago, IL) then submitted to the Genbank (accession number: KU 130124).

In addition, *T. danae* (accession no: FJ152165.1), *T. crenata* (accession no: FJ152164.1), *T. sima* (accession no: FJ152166.1), *T. admete* (accession no: FJ152163.1) and *T. prymna* (accession no: AM410537.1) included in the evolutionary analysis as performed in MEGA6 (Tamura et al., 2013). The Maximum Likelihood method based on the Kimura 2-parameter model (Kimura 1980) used to compute the evolutionary history and evolutionary distances by Maximum Composite Likelihood method (Tamura et al. 2012).

## Systematics

Family: Portunidae

Subfamily: Thalamitinae Paul’son, 1875

*Thalamita Latreille* Latreille, 1829

*Thalamita dana*e Stimpson, 1858

### Taxonomic history

*Thalamita crenata* (not Rüppell 1830) Dana 1852c: 282; Dana, 1855: pl. 17, Figure 7(a,b).

*Thalamita danae* (Stimpson, 1858) Stimpson, 1858a: 39; A. Milne-Edwards, 1861:366, pl. 30, Figure 1; Yu, 1979: 19: a–d. 68–69; Spiridonov, 1990. P. 73–74; Wee and Ng 1995: 73–82, Figure 37(A–C), 38(A–C), 39(A–C), 40(A,B), 41(A–D), 42(A–I); Davie 2002. (From, A. Milne Edwards 1861:366, pl. 30, Figure 1); Poore and Ahyong, 2004. 427–428, Figure 136 (a); Ng et al., 2008; Kumar and Wesley, 2010. P 4.

**Figure 1.**
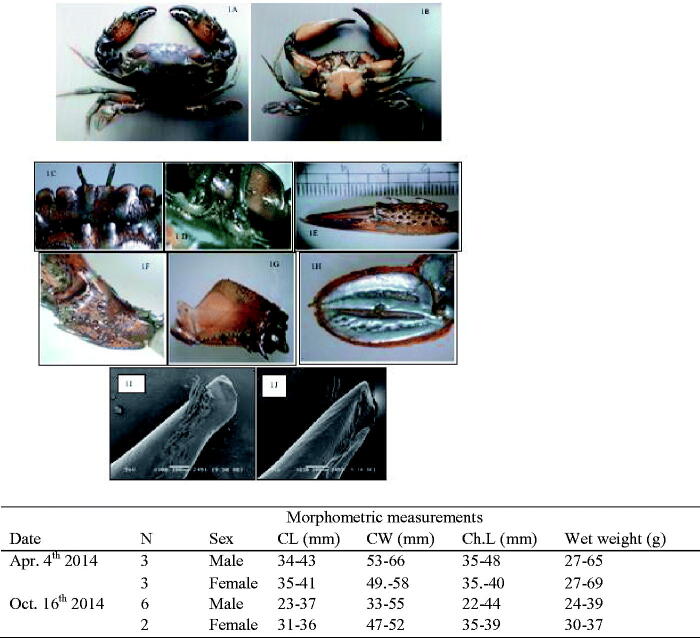
*Thalamita danae* (Stimpson, 1858): (A) dorsal view, (B) ventral view, (C) frontal region of male, (D) basal antennal article, (E) outer surface of right cheliped of male, (F) carpus of chelipeds, (G) menus of chelipeds, (H) dactylus of natatory leg, (I) apical part of right G1 (dorsal view) and (J) apical part of right G1 (ventral view) and morphometric measurements.

Type locality: Karachi, Pakistan.

New material: Three males, three females on 4 April 2014 and six males, two females on 16 October 2014 from Buleji (24° 47 815°N,67° 49 280°S) Karachi, Pakistan.

## Habitat

Intertidal areas of rocky shore and mudflats of sand or muddy sand with stones, areas with pebbles under stones.

## Colour

Whole body light and dark greenish coloration with slight purplish shade.

## Distribution

East Africa to tropical Indo-west Pacific, Australia, Taiwan, Mozambique, Red Sea, India, China, Japan, Singapore, Hong Kong, Philippines, Malaysia, Indonesia, New Caledonia, Marshall Islands, Fiji, Samoa, now from Pakistan.

## Morphological and molecular comparison

The present reported specimen of *T. danae* from the coastal waters of Pakistan compared with the collection of the

Natural History Museum London (BMNH 1986.848 a: 1%, R. Seed coll. 1986, Tolo Harbour, Hong Kong) and with neotype material of Wee and Ng (1995) as during the great Chicag fire,

Stimpson’s type material of *Thalamita danae* as was destroyed (Apel and Spiridonov 1998) and also with the material of Singapore: Labrador beach reef flat, August 1993: (ZRC) 1993.7358 deposit in Raffles Museum Treasure in Zoological Reference Collection (ZRC), National University of Singapore. After the molecular comparison; the present specimens showed 97% sequence similarity with the sequences of *Thalamita danae* voucher ULLZ 4760 16S rRNA gene, downloaded from Gene Bank (http://www.barcodinglife.org/) (accession no: FJ152165.1) specimen collected from Labradar, Singapore, in 23 December 1990 and deposit in University of Louisiana at Lafayette Zoological Collection (ULLZ) as a voucher no. ULLZ 4760. The specimen of *T. danae* confirmed from the identification keys of Yu (1979); Wee and Ng (1995); 73–82, Figures 37(A–C), 38(A–C), 39(A–C), 40(A,B), 41(A–D), 42(A–I); Poore and Ahyong (2004). 427–428, Figure 136a.

### Description

Carapace sub hexagonal or sub trapezoidal, markedly broader than long. These specimens distinguish by the fourth tooth of the Antero-lateral borders being rudimentary and sometimes even absent. Carapace densely pilose except on the teeth and raised transverse ridges. Frontal ridges smooth but distinct, proto gastric and mesogastric ridges with markedly granular outline. Epibranchials ridges interrupted by the metagastric ridge. The smooth ridge across cardiacs and each mesobranchial regions (Figure 1(a)). Front divided into six lobes, the median teeth shorter than the sub-medians, the sub-medians teeth partially overlap the median teeth with truncate anterior borders, separated by narrow notches (Figure 1(c)), Sub medians with inner border directed obliquely inwards and overlap laterals teeth as broad as sub-medians, anterior border bluntly round and separated from sub-medians by a V-shaped notch. Five anterolateral teeth all stout, first three sub-equal and similar, fourth and fifth smaller than those preceding . The orbital prolongation of basal antennal joint/segment wider than the major diameter of the orbit, with the granulated ridge (Figure 1(d)).

Chelipeds larger than ambulatory legs, bearing a set of spines on distal half of merus, several large granules on proximal half. Palm is armed with five spines, two on inner border, one on the middle of the outer boarder. Chela palm outer surface with three casts (Figure 1(e,f)). The natatory leg has a strong spine on the outer distal angle of the merus, the posterior margin of the propodus may have fine serrations and small spines numbering 4–11 may vary on the two side of the same individuals (Figure 1(g,h)). The first gonopod is sinuous with the opening apical, distal portion tapering off to a blunt oblique tip. Outer surface bears a terminal clump of bristles from which row of progressively shorter bristles runs down obliquely towards the outer edge of Gl. At the distal end of the terminal clump, as many as seven conical spines can be seen (Figure 1(i)). Basal lobes bluntly rounded (Figure 1(j)).

## Molecular results

Identification of *T. danae* based on the genetic similarity through BLASTn. The partial sequence of *T. danae* was obtained from gene bank accession no: FJ152165.1, showed 97% high sequence similarity, confirming non-specific status (Mantelatto et al. 2009) of type from Singapore, Labrador in 1990 and the new material *T. danae* from coastal waters of Pakistan.

**Figure 2.**
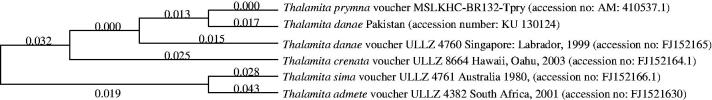
Molecular phylogenetic analysis of genus *Thalamita* by maximum-likelihood method Kimura 2-parameter model based on partial mitochondrial 16S rRNA gene (386 bp).

The evolutionary history was inferred using the maximum-likelihood method based on the Kimura 2-parameter model (Kimura 1980) and the result was expressed that the Partial sequence of *T. dana*e showed 95% similarity with *T. crenata* (Hawai, Oahu, 2003), 90% with *T. sima* (Australia, 1980), 93% with *T. admete* (Africa, 2001) and 86% with *T. prymna* with the evolutionary rate of 0.01 for base substitutions. The molecular datasets consisted of 516 aligned characters. The maximum composite-likelihood (MCL) approach showed that 0.015 distance between *T. danae* (Pakistan) and *T. danae* (Singapore) and 0.017 between *T. danae* and *T. prymna* (Figure 2).

## Taxonomic remarks

*Thalamita dana*e reported as new range based on morphological and molecular analysis of fresh material procured from coastal waters of Pakistan. The morphological identification described according to Poore and Ahyong (2004) and 97% similarity of 16S rRNA gene sequence (Mantelatto et al. 2009). The species morphologically more similar to *T. prymna* as reported earlier (Alcock 1899; Chhapgar 1957; Tirmizi and Kazmi 1996) but the *T. Prymna* specifically distinct due to absence of spines on the basal antennal segment joints (Kossmann, 1877). The much similar morphological characters indicated the need of identification of preserved specimens of previously reported *T. prymna* for species confirmation.
